# Prevalence of falls in elderly women

**DOI:** 10.1590/1413-78522015230300816

**Published:** 2015

**Authors:** Priscila Regina Rorato Vitor, Ana Carolina Kovaleski de Oliveira, Renan Kohler, Gabriele Regiane Winter, Cintia Rodacki, Maressa Priscila Krause

**Affiliations:** 1 Academic Department of Physical Education, Universidade Tecnológica Federal do Paraná, Curitiba, PR, Brazil

**Keywords:** Accidental falls, Aged, Physical fitness

## Abstract

**OBJECTIVE:**

To verify prevalence of falls and fear of falling, and to compare functional fitness among elderly women fallers and non-fallers.

**METHODS:**

Seventy-eight elderly women participated in this study. Cases of falls and the fear of falling were self-reported by the elderly women, while the functional fitness was measured by a set of functional tests. Mean and standard deviation were used to describe the sample. Independent t-test was used to compare functional fitness between groups.

**RESULTS:**

The prevalence of falls in this sample was 32.4%. Among women fallers, 40% self-reported a high fear of falling.

**CONCLUSION:**

It is recommended that functional and resistance exercises are included in the preventive strategies for reducing risk factors for falls and its determinants in elderly women. *Level of Evidence II, Prognostic-Prospective Study.*

## INTRODUCTION

Upon aging there is a progressive decrease in functional capacity, verified by reduced strength of lower limbs, impaired balance and agility. Such changes may impact a patient's daily activities such as climbing and descending stairs or siting and standing up from a chair, besides reducing the ability of the elderly to promptly respond to external disturbances, such as slipping and stumbling, and to restore balance.^1^ All these factors contribute to the increased risk of falls.^2^^-^ 5 Moreover, osteopenia and mainly osteoporosis are also considered among the factors that increase vulnerability to fractures from falls, especially the fracture of the femur.^6^

Individuals who have fallen can develop post-fall syndrome (classified as an emotional disorder), characterized by the fear of falling again.^7^^,^ 8 The fall, besides causing a decrease in mobility, can also cause permanent or temporary physical dependence because of slow rehabilitation process.^9^ Consequently, the elderly tend to restrict their activities due to disability, fear of falling and pain resulting from injury, further aggravating the functional decline, adversely affecting the quality of life.^6^

It is also noteworthy that the occurrence of falls and its treatment raises the public and individual budget. Data shows that the number of admissions increases every year, especially in women. In 2001 approximately 15,000 women were hospitalized, and in 2009, more than 20,000 admissions were registered.^6^ Such evidence shows the importance of analyzing the factors that influence falls and to compare the magnitude of functional changes. Whereas functional tests, which aim to reproduce the demands of daily life, have a higher correlation with the risk of falls than maximum strength tests, this study aims to determine the prevalence of falls and resulting fractures in elderly women, and to compare the functional fitness among women who have suffered falls or not in the last year.

## METHODS

**Figure 1.**
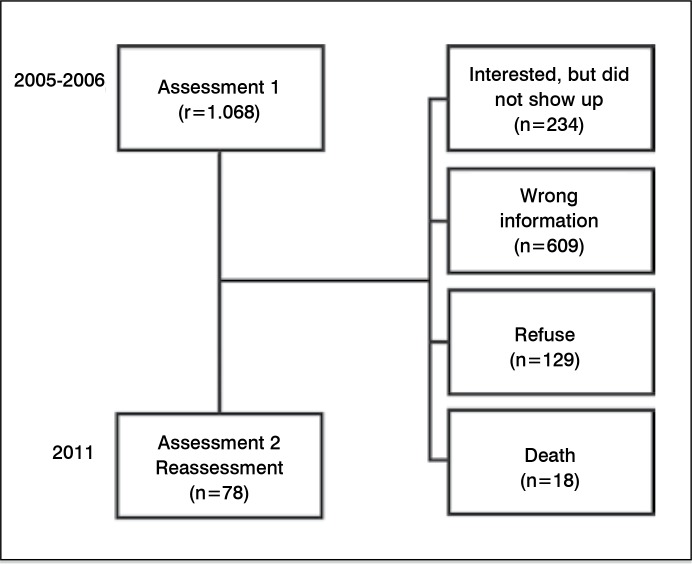
Assessment flow chart

This research is part of a longitudinal study, however, the data come only from the second evaluation. The variables evaluated in the first evaluation (2005-2006) were reevaluated (second evaluation) in the first half of 2011 - a mean interval of 5.8 years. (Figure 1)

This is a prediction-prospective, cross sectional study, being part of the Independent Senior Program. The first evaluation of the program began in 2005 and ended in 2006 with the participation of 1,068 elderly; the second assessment was conducted in 2011 with 78 participants. The initial contact with potential participants from the first evaluation was conducted via telephone by a trained member of the group of researchers who reported the purposes of this research, possible benefits and risks linked.

The protocol of the second evaluation was approved by the Ethics Committee of *Pontifícia Universidade Católica do Paraná, Brazil* (CEP No. 0004798/11) according to the standards established in the Declaration of Helsinki and Resolution 196/96 of *Conselho Nacional de Saúde* on research involving human subjects.

Before starting the data collection, a researcher asked the participant to sign the Free and Informed Consent, conditioning his/her participation on a voluntary basis. Participants who did not attend the second assessment (n=990) were excluded from the study.

The occurrence of falls reported by the elderly was assessed using the following question: "Did you suffer from a fall last year?" When an affirmative response was given, the participant was also questioned whether the fall caused any type of fracture or injury, in which anatomical site and, if the individual had "fear of falling again," and in this case the answer choices were: none, moderate or high fear.

Anthropometric variables (body mass and height, BMI, and waist circumference) were obtained according to procedures proposed by Lohman et al.^10^

Functional capacity was assessed by the following tests:

Walk test: it assesses the aerobic endurance. To this end, the maximum distance was determined on a six-minute walk along a rectangular path (18 x 9 m).^11^

Forearm flexion test: it assesses the upper limb strength. The maximum number of repetitions performed correctly by the rotational movement of the forearm (dominant limb) holding a 5 lb weight for 30 seconds.^11^

Sitting and rising from a chair test: it evaluates the lower limb strength. The maximum number of repetitions performed correctly to sit in and get up from a chair for 30 seconds.^11^

Trunk flexion test: it assesses the flexibility of lower limbs. To this end, we determined the maximum range obtained by performing the trunk flexion movement toward the tip of the foot (dorsiflexion), with overlapping hands, without flexing the knee.^11^

The 8-Foot Up-and-Go test: it assesses the agility and dynamic balance. To this end, we determined the best time that the participant obtained by performing the movement of rising from a chair, walk to a cone placed 2.44 m forward, and returned, sitting in the chair.^11^

Grip strength was measured by a digital dynamometer (Takei, Japan, 100 Kg capacity). To this end, the individual remained in standing position, holding the measuring instrument with the dominant hand and then the grip strength was measured.^12^

All the tests were performed twice (except for the walk test), always considering the best result achieved.

### Statistical Analysis

Analyses were performed using the Statistical Package for Social Sciences (SPSS 18.0). The descriptive analysis of the data was presented as mean and standard deviation. The independent t-Test was used to compare the functional fitness among women who have suffered falls or not in the last year (p <0.05).

## RESULTS

Among the participants, 32.4% suffered falls in the past year (Falls_1) and 67.6% did not (Falls_0). By comparing the functional aptitude among the groups it was noticed that none of these variables were significantly different. (Table 1) However, it was noticed that the elderly who did not fall had a higher isometric strength (handgrip test) and dynamic balance and agility (8-Foot Up-and-Go test).

**Table 1.** Comparison of health indicators and components of functional fitness between groups who have suffered falls or not in the last year.

	Falls_0 (n=52)	Falls_1 (n=25)
Age (years old)	73.1 (5.7)	73.2 (4.1)
Body mass (kg)	69.7 (11.8)	65.4 (12.7)
Body height (cm)	154.5 (6.99)	154.3 (6.83)
BMI (kg/m^2^)	29.1 (4.2)	27.3 (4.1)
Waist circumference (cm)	92.7 (11.4)	89.1 (10.5)
Trunk Flexion (cm)	2.09 (10.54)	4.04 (9.63)
Manual Dynamometry (kg)	23.4 (4.6)	21.9 (4.5)
Forearm flexion (rep)	11.8 (4.0)	11.6 (3.0)
Sit and reach chair (rep)	12.0 (2.8)	12.0 (3.0)
8 Foot-Up-and-Go (sec)	6.35 (1.94)	6.45 (1.73)
Walk test (m)	455.1 (91.9)	462.4 (97.1)

Falls_0: Participants who did not suffer any falls in the last year; Falls_1: Participants who suffered falls in the last year; kg: kilogram; cm: centimeter; sec: seconds; rep: repetitions; IBM: Body mass index.

**Figure 2.**
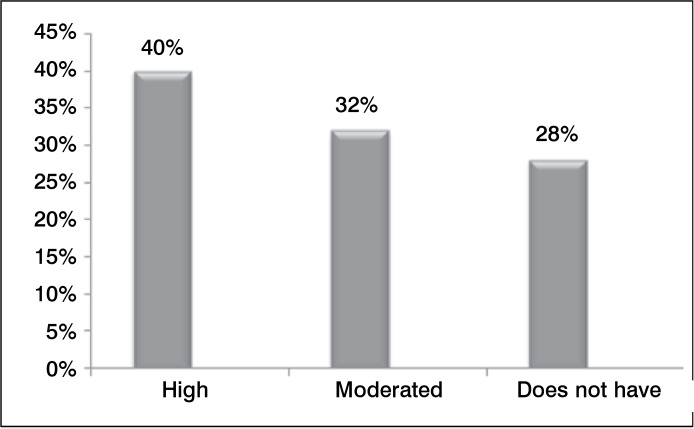
Relative frequency of fear of falling again.

Among women who had suffered falls, 10 elderly reported having high fear, eight moderate and only seven had no fear of falling again. Of the elderly women who reported having high fear of falling, one resulted in knee fracture and another an ankle fracture, while among women who reported moderate fear, there were two cases of right hand and foot fractures. (Figure 2)

## DISCUSSION

The prevalence of falls (32.4%) in this study was lower than other studies that evaluated independent and autonomous elderly participants of the Health Promotion Project in Brasilia (51.8%),^3^ elderly residents in zones covered by *Unidades Básicas de Saúde* (42.0%),^13^ as well as institutionalized elderly women in Pelotas, RS (37.3%),^14^ São Carlos, SP (76.6%),^2^ or institutionalized elderly men and women in Goiânia, GO (54.2%).^15^ It is noteworthy that the lower prevalence of falls was found in elderly men and women practitioners of a physical exercise program for approximately two years (22.2%),^16^ indicating that physical exercise can minimize the occurrence of falls in the elderly. The age of the elderly in this study was lower than the others studies, and it can explain the lower prevalence of falls that was found. According to Smith et al*.*,^13^ in a survey with a large elderly sample (n=4,003), the probability fall increases with advancing age, and it is about 29% greater than in "younger elderly" (65-70 years old).

Up to now few Brazilian studies have assessed the prevalence of falls together with the impact on functional fitness, making it difficult to compare the data. This research indicates that functional fitness did not differ significantly between women who have experienced falls or not in the last year, however the elderly who did not fall showed better results in isometric strength (handgrip test) and dynamic balance and agility (8-Foot Up-and-Go test). This was reported by authors^3^^,^ 5 that observed low capacity of functional aptitude tests to discriminate elderly with and without a history of falls. However, other studies have shown a relationship between the functional tests and the increased risk of falls in the elderly.^17^

Evidence indicates that elderly victims of falls have reduced functional fitness or mobility as compared to those who did not fall.^18^^,^ 19 There is evidence that older adults with lower trunk flexibility had a significant relationship with the number of falls,^18^ and that elderly victims of fall showed impaired balance and poor agility (they needed more time to complete the stand up and walk test).^18^^,^ 19

In turn, Streit et al.^20^ conducted a study with the same tests used in this study to evaluate the functional fitness of the elderly practitioners of physical exercises. The data indicated that only the strength of the lower limbs was associated with falls. Specifically, it has been shown that low force levels increased the risk of falls 2.66 times (Odds ratio: 95%, CI 1.15 - 6.15). However, the other components of functional fitness did not significantly related to the risk of falls. Ishizuka et al.,^5^ on the other hand, observed that muscle weakness was significantly associated with a greater frequency of falls, as well as those with lower functionality. Thus, it is understood that maintenance of muscle strength can minimize the risk of falling of the elderly. 

A program of physical exercise can be considered an effective strategy to reduce the risk of falls and also to prevent functional decline, since it aims to improve the ability to produce strength.^21^ To achieve this goal, it is recommended that the program includes exercises aimed to enhance the ability to rapidly generate torque, i.e., powerful movements and not only muscle strength. In fact, power training programs with the elderly found correlations with the improvement of the performance of functional tests and reducing the risk of falling, than in the resistance training program - aimed at improving strength resistance.^21^ However, isometric strength assessment do not seem to detect differences in peak torque among those who have suffered falls or not.^1^

Besides muscle strength, balance and agility should also be considered in the exercise programs. As reported by Gai et al.^3^ assessing functional indicators in independent elderly patients from the project *Promoção da Saúde dos Idosos de Brasília*, body balance is indirectly associated with the occurrence of falls. Similarly, balance showed impaired in elderly residents in Amparo, SP, Brazil.^4^

Other factors associated with falls in the elderly living in the community are related to old age, such as sedentary lifestyle, poor self-rated health and higher use of chronic medications;^13^ as well as women who report more than two falls in the last year (recurring) present as risk factors age, previous fracture, sedentary lifestyle, poor quality of life, diabetes mellitus and use of benzodiazepines.^22^ On the other hand, institutionalized elderly women have an increased frequency of falls, associated with back problems, rheumatism or using psychotropic drugs in the last month.^14^

Rebelatto et al.^2^ observed a higher incidence of falls among institutionalized elderly with lower isometric strength of the upper limbs (handgrip or grip strength), older and unable to watch television. In addition, the authors highlighted the following risk factors in the sample: mornings, outside the internal institutional environment, while walking, on cement floors, dry, after tripping over something, and be shod with slippers presented as risk factors for falls.

Although the results presented in this study do not indicate significant differences, we can observe a similar trend among the evidence highlighted so far, in which elderly who fell had decreased strenth capacity and balance. Additionally, it is noticed that the differences can be explained by several reasons, because the causes and risk factors for falls in the elderly are a combination of individual aspects, the nature of the task associated with the characteristics of the environment. Among these factors, we can point out that age is directly correlated to the risk of falls. Thus, the limitation of the results of this study can be linked to the age of the sample, i.e., tests applied may not have detected differences between the groups due to the participants be considered as "young" elderly, and these may be continually performing tasks similar to the tests in their daily activities, and these do not represent a challenge or an unexpected event.^19^ However, evidence was presented that functional tests are able to identify differences between the groups with and without a history of falls in the "older" elderly (> 80 years old), which have both sensory and neuromuscular systems more impaired.^23^

Finally, among the elderly who fell, 40% reported a high fear of falling again and only 28% had no fear. The fractures reported were in knee, ankle, right hand and foot. The fear of falling is a factor rated among elderly due to its influence on mobility, balance, risk and history of falls.^7^^,^ 9 ^,^ 16 Pinheiro et al.^22^ reported that over 60% of the elderly who fell developed this feeling, and about 30% of these have functional limitations to perform instrumental activities of daily living and recreation. It is also suggested that the fear of falling is higher among women, older seniors, and that such individuals may present loss of confidence and a greater degree of dependence. All of these factors combined contribute to the post-falling syndrome.^7^^,^ 8

It is clear that one of the consequences of senescence is a decrease in bone and muscle mass, which directly influence the neuromuscular fitness.^2^ Thus, it is understood that elderly, naturally with advancing age, become more vulnerable to falls, increasing also the risk of fractures, particularly those with osteopenia or osteoporosis.^7^^,^ 22 Individuals with these clinical findings are more prone to fractures, mainly in the femur, leading to a temporary or even permanent dependence.^7^^,^ 9 Consequently, the more traumatic this process, the higher the chance of the elderly developing fall syndrome, i.e. fear of falling again because of the restrictions and complications of the traatment.^7^^,^ 16 Thus, elderly can feel aversion to carry out the activities in which the event occurred, further decreasing their level of physical activity, increasing the degree of sedentary lifestyle, and therefore, further increasing the risk of falling again, featuring a vicious cycle.^6^^,^ 7

The main limitation of this study is linked to the sample size, yet this fact does not minimize the scientific and clinical relevance of the findings in order to demonstrate that the functional fitness of elderly women was not compromised by the event "fall". In general, we can see a trend of functional decline in women who fell, indicating that they can recover and continue to perform activities of daily living (ADLs) usually held before the event. This is an important argument for this population to not develop psychological problems and reduce their ADLs, a fact which negatively impact their performance capability. In addition, it is noteworthy that "young elderly" who fell may not yet have compromised their functional fitness, but there is evidence that "old elderly" who fell show a decline in their functional fitness. 

## CONCLUSION

The evidence highlighted show a trend that the elderly who experienced falls present less functional fitness, specifically related to muscle strength, balance and agility. Therefore, strategies need to be developed to improve these capabilities in order to prevent falls to occur as a result of this process, such as osteoporotic fractures. It is recommended that functional and resistance exercises are included in preventive actions in order to assist in the reduction of risk factors and determinants of falls in the elderly. 
